# Effects of high intensity intermittent training on lipid profile and blood glucose overweight/obese university students

**DOI:** 10.15649/cuidarte.2624

**Published:** 2023-03-31

**Authors:** Diana Paola Montealegre Suárez, Edna Paola Ramos González, Luisa Fernanda Romaña Cabrera

**Affiliations:** 1 . Fundación Universitaria María Cano. Grupo de Investigación FISIOTER. Neiva-Colombia. Email: dianapaolamontealegresuarez@fumc.edu.co Fundación Universitaria María Cano Fundación Universitaria María Cano Colombia dianapaolamontealegresuarez@fumc.edu.co; 2 . Fundación Universitaria María Cano. Neiva- Colombia. Email: ednapaolaramosgonzalez@fumc.edu.co Fundación Universitaria María Cano Fundación Universitaria María Cano Colombia ednapaolaramosgonzalez@fumc.edu.co; 3 . University Of Baltimore Maryland-USA. Email: Luisa.romanacabrera@ubalt.edu.co University Of Baltimore Maryland USA Luisa.romanacabrera@ubalt.edu.co

**Keywords:** High-Intensity Interval Training, Obesity, Lipids, Glucose., Entrenamiento Interválico de Alta Intensidad, Obesidad, Lípidos, Glucosa., Treinamento em Intervalo de Alta Intensidade, Obesidade, Lipídios, Glucose

## Abstract

**Introduction:**

High-Intensity Interval Training (HIIT) involves developing exercises in short periods of time with high intensity, followed by periods of rest between the series of exercises performed, and is considered an important tool to combat obesity. Therefore, the present work aimed to identify the effects of high-intensity intermittent training on the lipid profile in overweight and obese university students.

**Materials and methods:**

quasi-experimental study, consisting of a sample of 30 students, who were randomly assigned into two groups of 15 students, thus leaving a control group (continuous training): 15 and an experimental group (intermittent exercise of high intensity: 15. Moreover, there were carried out Laboratory tests before and after training to find the lipid profile. Also, the realization of 20 training sessions, which were distributed three times a week, with an average duration of 50 minutes. Additionally, the performed of statistical tests with a level of statistical significance of p <0.05.

**Results:**

there is a statistically significant relationship in the HDL value of the control and experimental group. The Glucose values show statistically significant relationships in the experimental group (p = 0.001).

**Conclusions:**

Intermittent high-intensity training and continuous moderate-intensity work generate a decrease in LDL and Triglycerides variables and an increase in HDL. However, they are not statistically significant after 20 training sessions. However, high- intensity intermittent training results in glucose-lowering in overweight and obese people.

## Resumen

**Introducción:**

El Entrenamiento Interválico de Alta Intensidad (HIIT) implica desarrollar ejercicios en períodos cortos de tiempo con alta intensidad, seguidos de períodos de descanso entre las series de ejercicios realizados, y se considera una herramienta importante para combatir la obesidad. Por lo tanto, el presente trabajo tuvo como objetivo identificar los efectos del entrenamiento intermitente de alta intensidad sobre el perfil lipídico en estudiantes universitarios con sobrepeso y obesidad.

**Materiales y métodos:**

estudio cuasi-experimental, conformado por una muestra de 30 estudiantes, quienes fueron asignados aleatoriamente en dos grupos de 15 estudiantes, quedando así un grupo control (entrenamiento continuo): 15 y un grupo experimental (ejercicio intermitente de alta intensidad: 15. Además, se realizaron pruebas de laboratorio antes y después del entrenamiento para determinar el perfil lipídico, así como la realización de 20 sesiones de entrenamiento, las cuales se distribuyeron tres veces por semana, con una duración promedio de 50 minutos, adicionalmente se realizó la pruebas estadísticas con un nivel de significación estadística de p < 0,05.

**Resultados:**

existe una relación estadísticamente significativa en el valor de HDL del grupo control y experimental, los valores de Glucosa muestran relaciones estadísticamente significativas en el grupo experimental (p = 0,001)

**Conclusiones:**

El entrenamiento intermitente de alta intensidad y el trabajo continuo de intensidad moderada generan una disminución de las variables LDL y Triglicéridos y un aumento de HDL. Estos datos, no son estadísticamente significativos después de 20 sesiones de entrenamiento. Sin embargo, el entrenamiento intermitente de alta intensidad da como resultado una disminución de la glucosa en personas con sobrepeso y obesidad.

## Resumo

**Introdujo:**

O Treinamento Intervalado de Alta Intensidade (HIIT) envolve desenvolver exercícios em curtos períodos com alta intensidade, seguidos de períodos de descanso entre as séries de exercícios realizados, sendo considerado uma importante ferramenta no combate a obesidade.

**Objetivo:**

identificar os efeitos do treinamento intermitente de alta intensidade no perfil lipídico de universitários com sobrepeso e obesidade.

**Materiais e métodos:**

estudo quase-experimental, composto por uma amostra de 30 alunos, que foram distribuídos aleatoriamente em dois grupos de 15 alunos, ficando assim um grupo controle (treinamento contínuo): 15 e um grupo experimental (exercício intermitente de alta intensidade: 15. Além disso, foram realizados exames laboratoriais antes e após o treinamento para determinado do perfil lipídico, além da realizado de 20 sessóes de treinamento, que foram distribuidas tres vezes por semana, com durado média de 50 minutos. Além disso, testes estatísticos com nivel de significancia estatística de p < 0,05.

**Resultados:**

há relado estatisticamente significativa no valor de HDL do grupo controle e experimental. Os valores de Glicose mostram relagóes estatisticamente significativas no grupo experimental (p=0,001).

**Condusdes:**

Treinamento intermitente de alta intensidade e trabalho contínuo de intensidade moderada geram diminuido das variáveis de LDL e triglicerídeos e aumento de HDL. Embora, eles nao sao estatisticamente significativos após 20 sessóes de treinamento. No entanto, o treinamento intermitente de alta intensidade resulta na redugao da glicose em pessoas com sobrepeso e obesas.

## Introduction

Obesity is classified as a multifactorial disease^1^, characterized by the accumulation of neutral fat in adipose tissue^2^ with a Body Mass Index equal to or greater than 30, typically responsible for morbidity and premature mortality^3^.

People with obesity (generally abdominal type) have atherogenic dyslipidemia. It is recognized by the increase in triglycerides and cholesterol levels associated with low-density lipoproteins (LDL-C) and decreases in the ranks of cholesterol linked to high-density lipoproteins (HDL-C4), as well as an increase in glucose and blood pressure^5^.

However, high concentrations of serum lipids cause pathologies such as arteriosclerosis since fats are stored in the walls of the arteries, reducing their elasticity, and reducing their diameter^6^.

For its part, the young population tends to adopt unhealthy lifestyles, such as a sedentary lifestyle and inappropriate foods, among others^7^. Likewise, due to hourly intensities, university students' lack of ease in acquiring low-calorie foods^8^, among other factors, has led to an increase in the consumption of foods rich in sugar, which are considered risk factors for the development of overweight or obesity and type II diabetes mellitus^9^.

Authors such as Louzada and Cols^10^ reveal that exercise plays an essential role in fat management. Therefore, High-Intensity Interval Training (HIIT) involves developing exercises in short periods with high intensity, followed by periods of rest between the series of exercises performed^11^^-^^12^ and is considered an important tool to combat obesity, as it increases the benefits of aerobic work in less time^13^.

Therefore, the present work aimed to identify the effects of high-intensity intermittent training on lipid and glucose profiles in overweight and obese college students.

## Materiales y Métodos

### Subjects

Quasi-experimental type study. The population corresponded to 106 students between the ages of 18 and 24 who attended the gym of the María Cano University Foundation, Neiva, during the second period of 2019. The sample was selected in a random probabilistic way using Microsoft Excel and corresponded to 30 students, according to the formula for finite populations.

### Inclusion and exclusion criteria

The students, including were active members at the María Cano gym during 2019, who signed the informed consent and presented a BMI Body Mass Index greater than 25 kg / m2 (overweight). Those students who did not complete all the assessment and training sessions were excluded.

### Ethics

The research follows resolution 008430 and the Declaration of Helsinki. Likewise, the ethics committee of the María Cano university foundation a university in Colombia approved the investigation, through the extraordinary session # 02 of 2019.

Initially, each of the research participants was made aware of the purpose, benefits, and risks. In the same way, the participant fills out the informed consent, which was used as support to verify the desire to be part of the research process.

### Procedures

The members of the sample were randomly assigned into two groups of 15 students, thus leaving a control group (continuous training): 15 and an experimental group (high-intensity intermittent training: 15. However, only 14 students received all intervention sessions. It is highlighted those various studies take samples of less than 40 people: 35 people^14^ 16 people^15^, 32 participants^16^, 24 participants^17^, 32 participants^18^ Each student voluntarily signed the informed consent, thus accepting participation in the research.

Each of the participants signed the informed consent, so after it, the pretest evaluation was performed on each participant, where laboratory tests were applied to find the lipid and glucose profile. Subsequently, 20 training sessions were used, distributed three times a week, with an average duration of 50 minutes. At the end of the total number of sessions, the post-test was applied to identify changes in the variables.

### Instruments

Lipid profile: to assess this variable, laboratory blood tests were applied to each of the participants before and after the intervention. The exams were carried out between 6 and 7 in the morning, with a previous fast> 12 h. HDL, LDL, Triglycerides, and VLDL values were analyzed.

Glucometry: To measure blood glucose, the Prodigy Auto Code lot # 107120005 brand glucometer with Prestige Suave lancets was used. All participants attended a fast from 8 to 12 hours between 6:30 and 7:30 a.m.

### Design and implementation of the control group intervention

All training sessions were carried out in an endless band, using a cyclical pattern that always started with a 10-minute warm-up speed of 6.5 Speed.

Later in the 30-minute core phase, intensity increases of two minutes were made at a speed of 8.5 Speed, followed by one minute with a speed of 13 Speed. Thus, these load increases were carried out three times during the core phase.

The cool-down phase lasted approximately 10 minutes under a speed of 5 and ended with 30-second static stretching of the upper and lower extremities.

### Design and implementation of the Experimental Group intervention

The experimental group started each training session with a 10-minute warm-up, either on the treadmill, bicycle, or jump rope.

The major phase was composed of 30-minute circuits with high intensity and short duration. Two circuits of 15 minutes each were worked with two minutes of recovery between each one.

Each circuit consisted of 5 to 6 exercises. Each participant performed as many repetitions as possible in the indicated time. These circuits' exercises contained burpees, semi-burpees, tabatas, loop jump, box jump, trot around the block, squat, jump squats, ABS plank, ABS in their different variations raising and lowering the box, Jump Jack, skipping. It ended with a 10-minute warm-up for a total of 50 minutes.

**Table 1:** Distribution of training sessions.

Load magnitude	Control group	Experimental group
Frequency		3 times per week
Intensity	6.5 speed 83. 8.5 speed x 2 minutes 84. 13 speed x 1 minutes	70 - 90%
Duration		Warm-up: 10 minutes Central phase: 30 minutes Cool down: 10 minutes
Training method	Continuous (enexercise treadmill) High intensity intermittent (Circuits)	

*Data analysis*

The data were entered into a matrix in Microsoft Excel and then exported to the statistical program (SPSS) version 24 where frequencies of all the variables for each group were generated. The database was stored in Mendeley Data^19^.

Likewise, was applied the Shapiro-Wilk test in order to identify whether the variables had a normal distribution. Also, was used the t-test to compare the quantitative parametric variables between the two groups. Finally, the Student's and in the case of comparison of non-parametric variables, the Wilcoxon test was used with a level of statistical significance of p <0.05.

### Financing

The research was funded by the María Cano University Foundation.

## Results

**Chart 1:**
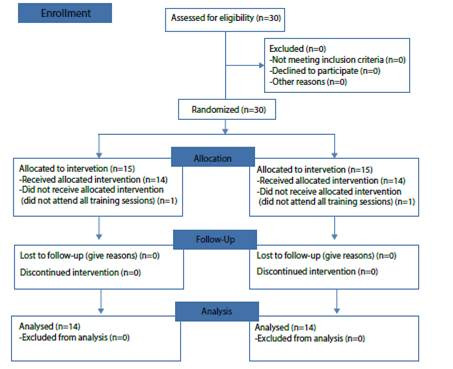

Thirty students participated equally distributed for the control and experimental group. However, two participants were excluded (one for each group) since they did not comply with all the intervention sessions. The patient flow chart is presented in Chart 1.

**Chart 2:**
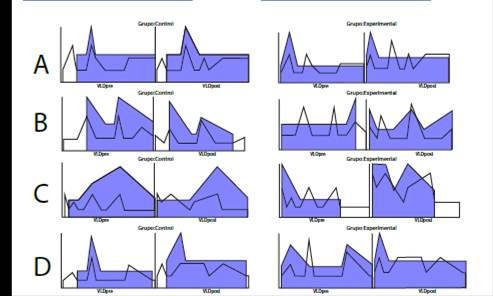
Comparison of variable lipid profile in control and experimental groups. Own elaboration.

Table 2 shows a statistically significant relationship in the HDL value of the control and experimental group (p = 0.004). However, the two groups presented decreases in LDL and triglyceride values after applying 20 intervention sessions (see Chart 2). Regarding the Glucose values, were found statistically significant relationships in the experimental group (p = 0.001).

**Table 2:** Changes in lipid profile and glucose after 20 intervention sessions.

Variables	Control group 107. 108. 109.				P	Experimental group 112.		P
	Pretest	Postest		Pretest	Postest	
	(n:14)	(n:14)		(n:14)	(n:14)	
VLDL	21.75 ± 7.59	22.25 ± 7.71	.78	19.50 ± 8.91	19.90 ± 9.05	.53
HDL	44.12 ± 6.33	45.25 ± 6.20	.16	42.30 ± 9.29	44.20 ± 9.11	.00
LDL	110.00 ± 25.80	109.75 ± 29.50	.09	92.50 ± 20.81	87.00 ± 21.37	.08
Triglycerides	114.71 ± 40.71	109.00 ± 38.20	.12	105.50 ± 47.60	100.30 ± 45.26	.72
Glucose	97.37 ± 8.20	97.10 ± 7.46	.04	101.373.90	97.40 ± 5.30	.00

**Chart 3:**
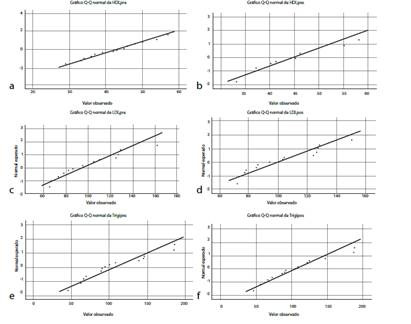
Distribution of the lipid profile variables before and after the intervention.

The median and interquartile ranges show better results in the variables HDL, LDL, and Triglycerides in the experimental group and less variability concerning the control group in the pretest and post test (Chart 2 and Chart 3).

*(a): HDL pretest distribution. (b): Posttest HDL distribution. (c): Pretest LDL distribution. (d): Post-test LDL distribution. (e): Pretest Triglycerides distribution. (f): Post-test triglycerides distribution.*

## Discussion

The objective of this work was to identify the effects of high-intensity intermittent training on the lipid profile in overweight and obese university students, where statistically significant changes were found in the HDL variable, both in the control and experimental groups. Likewise, the variables LDL and Triglycerides showed a decrease in their values, although these were not statistically significant.

These data are similar to an investigation carried out by Louzada and Cols^10^. They revealed that high- intensity intermittent training is more effective than continuous work to improve the lipid profile since it significantly reduces cholesterol and triglyceride levels and increases significant high-density lipoprotein levels during 60 days of training.

However, these data differ from those by Sabogal^20^, who reveals that he did not find statistically significant differences in biochemical markers in his studies. After the intervention in the group with high-intensity intermittent training and continuous training, a more significant reduction in the lipid profile is highlighted in people who performed continuous exercise of moderate intensity.

A study carried out by Sañudo and Cols^21^ reveals that a HIIT training combined with vibration in the rest processes and a hypocaloric diet can improve the global lipid profile in comparison with a HIIT training with diet or hypocaloric diet exclusively in overweight individuals or obesity.

On the other hand, an investigation carried out by González and Cols^4^ reveals that young university students have problems of excess weight and alterations in the lipid profile, which can trigger future damage to the coronary arteries due to increases in levels of LDL.

Moreover, Hernández and Cols^22^ mentioned that the treatment of atherogenic dyslipidemia is based on weight loss and physical exercise. Likewise, authors such as Laécio and Evaristo^23^ refer that physical exercise generates a decrease in the concentration of triglycerides (TG), low-density lipoprotein (LDL), and total cholesterol (TC). For its part, a study by Molina^24^ indicates that after 12 high-intensity intermittent training sessions, a significant decrease in body fat occurs in overweight and obese people.

Additionally, it was found that the glucose had a statistically significant improvement in the values of the experimental group, which performed high-intensity intermittent training. These data coincide with authors such as Flockhart and Cols^25^, who reveal that physical exercise has a positive effect on metabolic health related to glucose regulation after frequent training with high loads, where a reduction in intrinsic mitochondrial function can be observed that coincides with an alteration in glucose tolerance and insulin secretion.

It can be considered a limitation, not having control of the diet of the study population, since this can directly impact the variables evaluated. Likewise, the genetic aspects, the additional exercise routines and the sample size can be considered as limitations of the study.

## Conclusions

Intermittent high-intensity training and continuous moderate-intensity work generate a decrease in LDL and Triglycerides variables and an increase in HDL. However, they are not statistically significant after 20 training sessions. High-intensity intermittent training results in glucose-lowering in overweight and obese people. Therefore, it is suggested to carry out new studies with a greater number of sessions and in different population groups.
